# Task-dependent functional organizations of the visual ventral stream

**DOI:** 10.1038/s41598-019-45707-w

**Published:** 2019-06-27

**Authors:** Han-Gue Jo, Junji Ito, Barbara Schulte Holthausen, Conrad Baumann, Sonja Grün, Ute Habel, Thilo Kellermann

**Affiliations:** 1 Department of Psychiatry, Psychotherapy and Psychosomatics, Medical Faculty, RWTH Aachen University, 52074 Aachen, Germany; 2 JARA-Institute Brain Structure Function Relationship (INM-10), Research Center Jülich and RWTH Aachen University, 52074 Aachen, Germany; 3 Institute of Neuroscience and Medicine (INM-6) and Institute for Advanced Simulation (IAS-6), Research Center Jülich, 52425 Jülich, Germany; 4 Median Klinik Mecklenburg, 19217 Rehna, Germany; 5 Theoretical Systems Neurobiology, Faculty I, RWTH Aachen University, 52056 Aachen, Germany

**Keywords:** Object vision, Cognitive control

## Abstract

The visual hierarchy of the ventral stream has been widely studied. However, it remains unclear how the hierarchical system organizes its functional coupling during top-down cognitive process. The present fMRI study investigated task-dependent functional connectivity along the ventral stream, while twenty-eight participants performed object recognition tasks that required different types of visual processing: i) searching or ii) memorizing visual objects embedded in natural scene images or iii) free viewing of the same images. Utilizing a seed-based approach that explicitly compared task-specific BOLD time-series, we identified task-dependent functional connectivity of the visual ventral stream, demonstrating different correlation structures. Searching for a target object manifested both correlated and anti-correlated structures, separating the visual areas V1 and V4 from the posterior part of the inferior temporal cortex (PIT). In contrast, the ventral stream structure remained correlated during memorizing objects, but increased the correlation between the right V4 and PIT. On the other hand, V1 and V4 showed task-dependent activation, whereas PIT was deactivated. These results highlight the context-dependent nature of the visual ventral stream and shed light on how the visual hierarchy is selectively organized to bias object recognition toward features of interest.

## Introduction

The visual ventral stream is a series of hierarchical processing stages from the primary visual cortex V1 to inferior temporal cortex IT, in which neural interactions along this hierarchy enable us to recognize visual objects^1,2^. However, given its complex and diverse connectivity, it is difficult to describe the functional network, particularly when top-down cognition is involved. The ventral stream requires different functional properties of the hierarchy to incorporate visual features of interest into object recognition^3,4^, which may alter the functional coupling of the visual hierarchy according to task-goals. To address this hypothesis, here we examine task-dependent functional connectivity of the ventral stream, utilizing a seed-based approach investigating task-specific functional correlations of BOLD time-series. In order to derive the ventral stream, we conducted visual tasks that required distinct cognitive processes for object recognition: searching for a target object or memorizing objects embedded in natural scene images as well as free viewing of the same images.

Visual search requires coordination of information processing^3,5^. It tunes the functional properties of neurons to form a perceptual field based on prior knowledge about target-defining features^3^, in which each processing stage of the visual hierarchy contributes complementary information. The early stage V1 could provide specific information of simple visual features such as edge and contrast, while the intermediate stage V4 could contribute to more complex features like color, texture, and shape^6–8^. In contrast to visual search, memorizing objects demands deeper encoding processes that store information into memory. Since memory-related neural activation is feature-selective in the ventral stream^9,10^, the hierarchy system has also been suggested to play a significant role in memorizing objects^11–14^. The rationale behind this view is that if visual perception emerges via the hierarchical system, then the hierarchical process might also be crucial for storing visual information into memory. Together these aspects provide an essential link between the higher cognitive processes and the functional hierarchy of the ventral stream. However, it remains unclear how the ventral stream configures its functional structures according to the task goals.

To provide insight into this question, BOLD time-series in individuals performing visual cognition tasks were examined. A seed-based analysis was performed along the visual ventral stream, with the first cortical processing stage V1 subjected as the initial seed. Voxel clusters that revealed significant task effect on BOLD time-series correlation with the seed were identified as the regions of interests (ROIs) and these ROIs were further subjected as seeds for the subsequent seed-based analyses. We then show to which extent task-dependent connectivity strengths across the identified ROIs increases or decreases during each of the visual search, memory, and free view condition.

## Materials and Methods

Thirty-two right-handed volunteers took part in the study. All subjects had normal or corrected-to-normal visual acuity and were free from any history of neurological or psychiatric diseases. Written informed consent was collected from each subject after the procedure was fully explained. The study was approved by the ethics committee of RWTH Aachen University Hospital and carried out in accordance with the relevant guidelines and regulations. Data of four subjects were discarded from the analysis due to artifacts and technical failure during recordings. As a result, twenty-eight subjects (mean age = 23.86, SD = 3.36; 15 females) were included in the present report.

**Figure 1:**
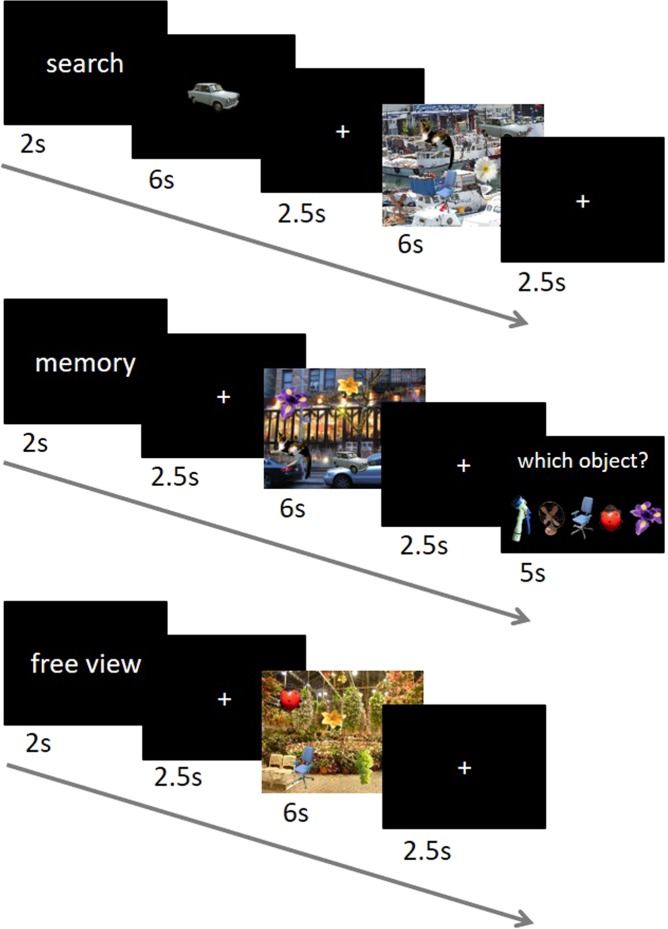
Sequence of events in each task condition (modified from^15^). The time course of each condition is shown in seconds. For the search condition, subjects were required to search the scene for a target object, while during the memory condition they memorized all objects embedded in the scene. No specific instruction was given for the free view condition. Note that the objects embedded in natural scene images are enlarged and randomly located for illustration purpose.

Task design, data acquisition, and behavioral results of this study have been reported in detail previously^15^, but the fMRI data presented here have not been reported in the previous study. In brief, subjects performed voluntary visual exploration of natural scene images under three different task conditions: searching for a target object, memorizing objects, and free viewing of the same images (Fig. 1). Each subject performed 30 trials for each task condition, resulting in a total of 90 trials. The order of the trials of the three different conditions was pseudo-randomized to avoid the same condition three times in a row. Each trial began with a 2 s display of one of three task indicators (“search”, “memory”, or “free view”). For the search condition, a target object was first displayed for 6 s after the task indicator and then a scene image was presented for 6 s. Subjects had to search the scene for the target object (every scene image contained five different objects; see below) and had to press the middle button of a three-response button device as soon as they found the object. In contrast, for the memory condition, a scene image was first presented for 6 s after the task indicator and subjects were required to find and memorize all five objects embedded in the scene image. Afterwards, they were presented with a probe array consisting of five objects with a cursor over one of them, among which only one object had been presented in the scene image. Subjects were given 5 s for a response by pressing the left and right buttons for moving the cursor to the left and right, respectively, and pressing the middle button for selection. For the free view condition, a scene image was presented for 6 s after the task indicator and subjects viewed the scene freely without any specific task. In all three conditions, a fixation-cross was presented for 2.5 s before and after the scene image presentation. The same fixation-cross was also presented for a random interval of 5.5–8 s between trials.

The scene image stimuli for the three task conditions were generated by placing object images on background images of natural scenes. For the objects, we draw 39 object images from the Microsoft image gallery and resized them (keeping the aspect ratios) to a size of on average 143.56 × 141.10 pixels. The objects consisted of flowers, animals, insects, fruits, furniture, tools, and transports. For the background, we collected 15 natural scene images of 1920 × 1200 pixels, eight of which were pictures taken by one of the co-authors and the rest were taken from the Internet. The images were photos of plants, flowers, fruits, gardens, boats, rooms, streets, and churches (details are available on request to the authors). The objects naturally blended into the background based on a contrast map for every pair of object and background images^15^ (see Supplementary Information for an example of the visual stimulus). A total of 90 stimulus images were generated based on 30 combinations of 15 backgrounds times two sets of five objects; each combination served for three variants of a stimulus image which differed only in the object positions. We used each of the three variants for each of the three task conditions, assuring that each and every combination appeared only once in each task condition.

The presentation of the scene images (background plus five placed objects) for each trial was implemented using the Presentation software (Neurobehavioral Systems, USA). The images were presented on a monitor screen (BOLDscreen, Cambridge Research Systems Ltd) at a refresh rate of 60 Hz. The images were displayed with a 1920 × 1200 pixels resolution, subtending 25.4 × 16.0° of visual angle.

### fMRI data acquisition and analysis

Participants were placed in a 3 Tesla MR scanner (Siemens MAGNETOM Prisma®, Siemens Medical Systems, Germany) with their right fingers positioned on a three-response button device. Before the main task, a 4 minutes magnetization-prepared rapid acquisition gradient echo image (MPRAGE) T1-weighted sequence was used to acquire structural images (TR = 1900 ms, TE = 2.52 ms, matrix = 256 × 256, 176 slices, voxel size = 1 × 1 × 1 mm^3^, flip-angle 9°). During the main task, functional imaging was performed using echo-planar imaging sensitive to BOLD contrast (voxel size: 3 × 3 × 3 mm^3^, 64 × 64 matrix, 36 slices, TR 1950ms, TE 30 ms, flip-angle 76°). MR-compatible EEG and eye tracking systems were also used to record physiological responses, which have been reported elsewhere^15^ and were not used in this study.

For preprocessing, SPM12 toolbox was used (https://www.fil.ion.ucl.ac.uk/spm/software/spm12/). Six motion parameters have been estimated and used to realign the functional images to their mean and the differences in image acquisition time between slices were corrected. The resulting images were co-registered to the structural image that was segmented into tissue components (gray and white matter, and cerebrospinal fluid), and normalized to the standard brain template from the Montreal Neurological Institute (MNI). Functional images were further smoothed using an 8 mm Gaussian kernel.

We first performed seed-based connectivity analyses using the functional connectivity toolbox CONN v.18^16^. The CONN toolbox computed Fisher-transformed correlation coefficient (z) between the average BOLD time-series of a given seed region and every voxel in the brain (seed-based correlation map). Before computing correlations, functional images were denoised, following the strategy implemented in the CONN toolbox; Images of the whole recording session were high-pass filtered at 0.008 Hz. Each delta function of the conditions was convolved with the canonical hemodynamic response function implemented in SPM12 and then entered as regressor in the general linear model (GLM). BOLD signal from the white matter and cerebrospinal fluid as well as functional outlier and motion parameters were taken as confound effects and regressed out. Besides, the BOLD signal changes associated with the presence or absence of task conditions were also regressed out. The BOLD time-series was then divided into scans associated with each block of task condition (task-specific BOLD time-series).

**Figure 2:**
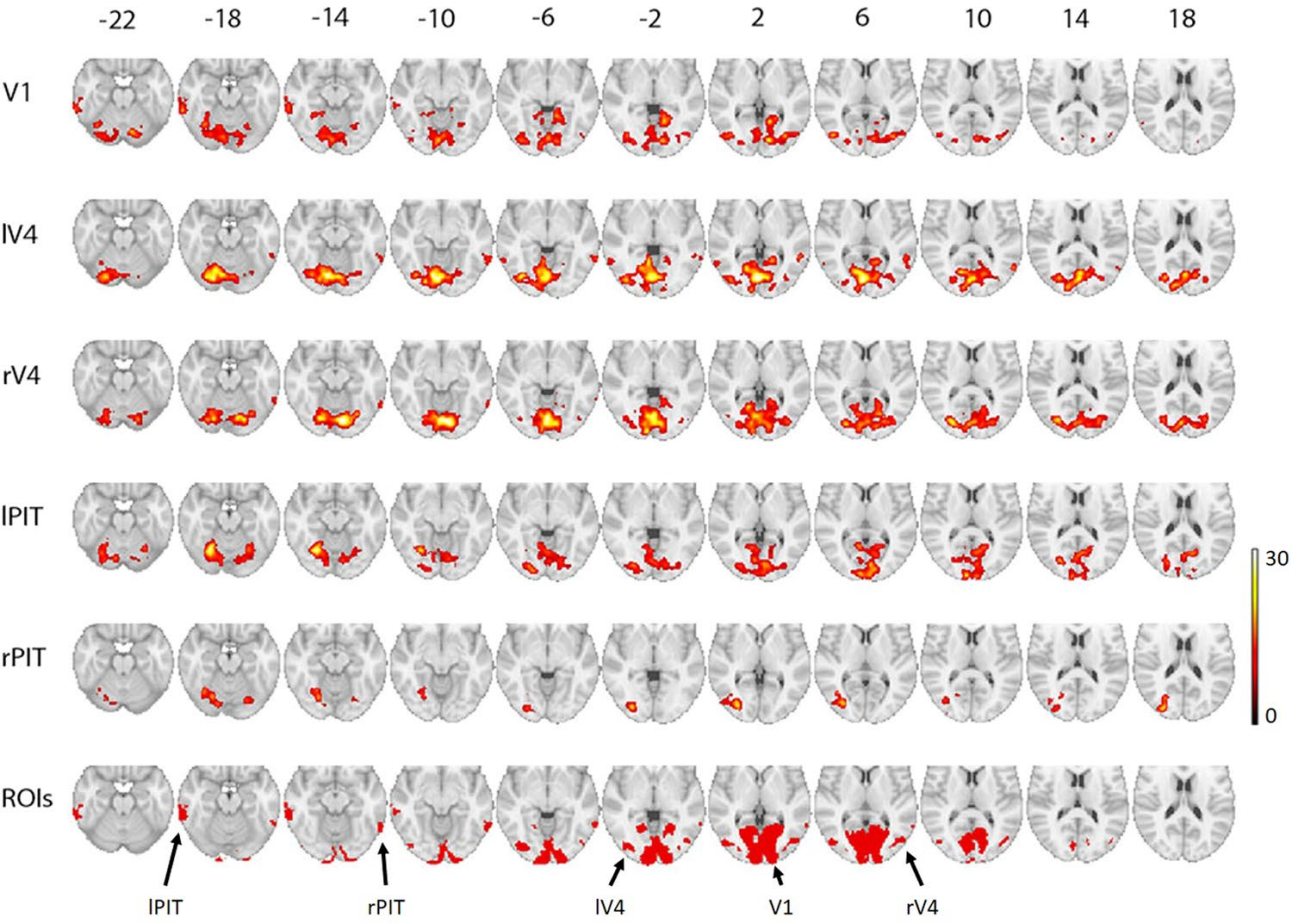
Task-dependent seed-based correlation maps of the visual ventral stream. Row-wise group level F-maps representing seed-based analysis show significant task-dependent correlations with a seed region indicated on the left column. The numbers on top indicate z coordinate in MNI space. The top five maps are presenting with threshold of uncorrected p < 0.001 at voxel level and FWE-corrected p < 0.05 at cluster level. On the basis of the seed-based maps (a detailed description is provided in the Results section), five ROIs were identified as shown on the bottom row. V1 = primary visual cortex; rV4 = right V4; lV4 = left V4; lPIT = left posterior part of the inferior temporal cortex; rPIT = right posterior part of the inferior temporal cortex. The peak MNI coordinates of the significant voxel clusters are shown in Table 1.

We defined the area reflecting the first stage of visual cortical processing as a seed (the primary visual cortex V1) based on an anatomical reference obtained from the Anatomy toolbox^17^ (the bilateral hOC1; see also the bottom row in Fig. 2), which overlapped with the BOLD signal responses from the conjunction map of the three task conditions (see Supplementary Information). The correlations between the mean BOLD time-series of the voxels in the V1 seed and the time-series of each voxel throughout the whole brain were then computed for each subject, separately for each task condition. The resulting correlations were subsequently used for group level analysis with a repeated measures ANOVA to investigate any differences across the three task conditions. Significance of the task effect was thresholded using a voxel level uncorrected p < 0.001 and cluster-level FWE-corrected at p < 0.05. The resulting voxel clusters in the ventral stream were identified as ROIs and subjected as seeds for the subsequent seed-based analyses. This seed-based approach resulted in five ROIs (see the Results section).

Next, we examined the interaction between the identified ROIs (ROI-to-ROI analysis). The average BOLD time-series was computed across all the voxels within each ROI. Fisher-transformed correlation was then computed for each pair of ROIs and subjected to a repeated measures ANOVA with task condition as a within-subject variable. Mean ± standard deviation of the mean were used to express the variables.

Lastly, to assess the BOLD signal changes in each ROI as a response to the scene images (the main effects of task-driven activation instead of correlation between regions), the parameter estimates reflecting the signal change for each condition versus baseline were calculated in the context of the GLM using SPM12. Six motion parameters and the presentations of the instruction, target object, and the probe array were modeled as regressors of no interest. The ROI activity was then extracted from these contrast estimates of each task condition.

## Results

### Behavioral results

For the search condition, mean performance across subjects was 46.6% (SD = 0.20) correct responses for detecting a target object. On the correct trials, mean response time was 3.0 s (SD = 0.36). For the memory condition, the mean performance was 48.0% (SD = 0.12) correct responses to a five-choice probe request of trials, where the chance level of choosing the correct object was 20%. These results ensure cognitive engagement of the subjects during the task conditions. Besides, our preceding study demonstrated different eye-movement behaviors between the conditions and further supported task-dependent cognitive processes^15^.

### Seed-based analysis

To identify task-dependent functional network of the visual ventral stream, we conducted a series of seed-based analyses. First, we defined the area reflecting the first stage of visual cortical processing as a seed (the primary visual cortex V1). The top row in Fig. 2 displays the map of the voxels with any significant task effects on their BOLD time-series correlations with the V1 seed. This map revealed significant task-dependent correlations with a widespread voxel cluster covering the early visual areas (V1, V2, and V3) and the intermediate visual area V4 as well as with a voxel cluster of the higher-order visual area, the posterior part of the IT (PIT). To obtain the correspondence area V4, the map was combined with an anatomical map of the ventral extrastriate and lateral occipital cortices (the bilateral hOC4d, hOC4la, and hOC4lp)^17^. The significant voxels within the anatomical map were defined as left and right V4 respectively (lV4 = 338 voxels; rV4 = 215 voxels; the bottom row in Fig. 2). It also revealed a significant task-dependent correlation with a voxel cluster in the left PIT (lPIT = 330 voxels), covering the posterior portion of the left inferior and middle temporal gyri. The observed V1 seed-based map thus demonstrated task-dependent correlations of V1 – lV4, V1 – rV4, and V1 – lPIT in the visual ventral stream.

Likewise, the extracted lV4 and rV4 were separately subjected to the same seed-based analysis. The lV4 seed-based map revealed a significant task-dependent correlation with a cluster covering the early visual areas and the rV4 (the second row in Fig. 2). Similar pattern of results was found for the rV4 seed-based map (the third row in Fig. 2), showing significant task-dependent correlations with the early visual areas and lV4. Notably, both the lV4 and rV4 seed-based maps identified the right PIT, covering the posterior portion of the right inferior and middle temporal gyri (rPIT). The rPIT was thus defined by the joint effect of the lV1 and rV4 seed-based maps (rPIT = 195 voxels).

Lastly, the BOLD time-series extracted from the lPIT and rPIT were separately subjected to the same seed-based analysis above. The lPIT seed-based map revealed a significant task-dependent correlation with a cluster covering the early visual areas, lV4, and rV4 (the forth row in Fig. 2). On the other hand, the rPIT seed-based map identified the lV4 (the fifth row in Fig. 2).

Taken together, this seed-based approach converged into five ROIs in the visual ventral stream (the bottom row in Fig. 2), representing a task-dependent connectivity network. However, we note here that there were also other significant voxel clusters (see Table 1), for instance, in the angular gyrus of the V1 and rV4 seed-based maps and in the frontal pole of the lV4 seed-based map, which are not located in the visual ventral stream.

**Table 1:** Peak MNI coordinates of significant voxel clusters.

Seed-ROI	Region	Cluster size	MNI			Z score
			x	y	z	
V1	Lingual Gyrus	3737	14	−58	0	5.47
	Intracalcarine cortex		12	−82	2	5.23
	Cerebellum Right VI		12	−72	−22	5.08
	Inferior Temporal Gyrus, posterior division	330	−64	−42	−18	4.85
	Middle temporal gyrus, posterior division		−68	−26	−14	3.78
	Inferior Temporal Gyrus, posterior division		−58	−28	−22	3.43
	Lateral occipital cortex, inferior division	512	−44	−76	6	4.83
	Lateral occipital cortex, inferior division		−38	−76	−2	4.48
	Lateral occipital cortex, inferior division		−38	−84	−6	4.30
	Cuneal cortex	208	−6	−84	32	4.50
	Cuneal cortex		−6	−80	24	4.13
	Cuneal cortex		−10	−88	26	3.92
	Supramarginal gyrus, posterior division	1388	−62	−48	26	4.47
	—		−42	−46	28	4.43
	Angular gyrus		−46	−58	30	4.41
Left V4	Lingual Gyrus	6587	−4	−78	2	6.38
	Lingual Gyrus		−12	−76	−12	6.02
	Lingual Gyrus		−4	−80	−10	5.86
	Supramarginal gyrus, posterior division	1657	−58	−46	40	5.38
	Postcentral gyrus		−58	−26	44	5.01
	Postcentral gyrus		−46	−34	58	4.64
	Middle temporal gyrus, temporooccipital part	331	54	−50	4	4.77
	Lateral occipital cortex, inferior division		56	−66	10	4.67
	Middle temporal gyrus, temporooccipital part		66	−52	−8	4.43
	Precentral gyrus	124	52	12	36	4.66
	Middle frontal gyrus		40	12	38	3.19
	Frontal pole	251	−42	42	−12	4.52
	Frontal pole		−40	46	−4	4.30
	Frontal pole		−34	38	−12	4.28
	Supramarginal gyrus, posterior division	248	48	−44	46	4.45
	Supramarginal gyrus, anterior division		50	−32	54	4.19
	Frontal pole	134	46	44	14	4.24
	Frontal pole		52	36	10	3.44
	Frontal pole		44	42	4	3.19
	Middle temporal gyrus, temporooccipital part	101	−58	−58	2	3.78
Right V4	Lingual gyrus	6596	8	−80	−12	6.58
	Lingual gyrus		−2	−76	−4	6.04
	Occipital fusiform gyrus		−22	−78	−16	5.55
	Supramarginal gyrus, posterior division	215	52	−38	48	4.78
	Supramarginal gyrus, posterior division		56	−40	32	4.03
	Supramarginal gyrus, posterior division		48	−46	50	3.94
	Supramarginal gyrus, posterior division	209	−52	−48	32	4.59
	Angular gyrus		−40	−56	34	4.00
	Supramarginal gyrus, posterior division		−60	−42	40	3.70
	Inferior temporal gyrus, temporooccipital part	88	62	−58	−12	4.31
	Inferior temporal gyrus, temporooccipital part		60	−50	−18	3.70
	Postcentral gyrus	83	−60	−26	40	3.82
	Supramarginal gyrus, anterior division		−56	−30	46	3.80
Left PIT	Temporal occipital fusiform cortex	5029	−26	−64	−16	6.05
	Occipital pole		−8	−96	8	5.28
	Precuneus cortex		12	−68	20	4.95
	Lateral occipital cortex, superior division	158	22	−82	26	5.80
	Supramarginal gyrus, anterior division	350	−62	−38	30	4.55
	Postcentral gyrus		−62	−24	44	4.31
	Supramarginal gyrus, anterior division		−56	−38	36	3.97
	Supramarginal gyrus, anterior division	195	66	−30	32	4.24
	Supramarginal gyrus, posterior division		58	−36	36	3.25
	Supramarginal gyrus, anterior division		60	−26	36	3.18
Right PIT	—	514	−32	−78	4	5.35
	Lateral occipital cortex, inferior division		−40	−64	6	4.51
	Lateral occipital cortex, inferior division		−38	−76	12	4.23
	Lateral occipital cortex, superior division	321	−28	−84	20	5.32
	Lateral occipital cortex, superior division		−28	−74	20	4.63
	Intracalcarine cortex		−22	−66	12	4.03
	Temporal occipital fusiform cortex	442	−34	−62	−18	4.86
	Occipital fusiform gyrus		−24	−70	−16	4.84
	Cerebellum left VI		−16	−80	−18	4.63
	Supramarginal gyrus, anterior division	325	−54	−32	52	4.40
	Postcentral gyrus		−66	−18	36	4.34
	Supramarginal gyrus, anterior division		−60	−40	40	4.00
	Cerebellum right VI	90	22	−74	−18	4.29
	Occipital fusiform gyrus		30	−76	−18	3.79
	Supramarginal gyrus, anterior division	84	52	−28	44	4.15

The brain regions of significant voxel clusters were identified based on an anatomical reference obtained from the Anatomy toolbox^17^.

### ROI-to-ROI analysis

**Figure 3:**
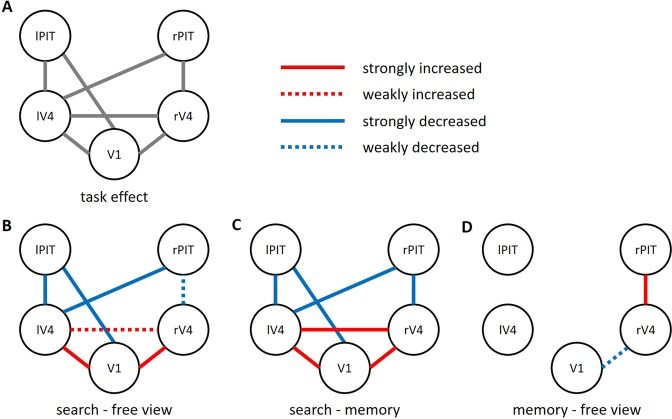
Task-dependent functional network identified by a seed-based approach. Relative changes in ROI-to-ROI correlations across three task conditions are shown. (**A**) Seven correlations out of ten possible pairs revealed significant task effects (p < 0.05, corrected). (**B**) During the search condition, the correlation strength across V1, lV4, and rV4 increased, while correlations of lPIT or rPIT with those of other visual areas decreased. Significant changes in correlations were indicated as a strong (p < 0.05, corrected) or weak (p < 0.05, uncorrected) change. The decreased correlation V1 – lPIT during the search condition revealed an anti-correlation (negative or inverse correlation) across subjects (Fisher-transformed z = −0.117 ± 0.161; one-sample t-test, p < 0.001). (**C**) This pattern of connectivity structure was consistent when the search condition was compared with the memory condition. (**D**) In contrast, the memory condition, the correlation rV4 – rPIT increased, while V1 – rV4 decreased. V1 = primary visual cortex; rV4 = right V4; lV4 = left V4; lPIT = left posterior part of the inferior temporal cortex; rPIT = right posterior part of the inferior temporal cortex.

For further validation of the identified network, we examined the interactions between the BOLD time-series averaged within each of the five ROIs. A repeated measures ANOVA showed a significant main effect of task condition in seven correlations out of ten possible pairs. Figure 3A shows the ROI-to-ROI correlations that yielded a significant task effect (p < 0.05; Bonferroni corrected for 10 multiple tests).

To directly test the task effect to which extent the correlations increase or decrease during each of the three task conditions, we conducted post-hoc tests for each of the seven ROI-to-ROI correlations (p < 0.05, Bonferroni corrected for 7 × 3 multiple tests). Relative to the free view condition, the search condition showed increased correlations for V1 – lV4 (free view, z = 0.299 ± 0.153; search, z = 0.485 ± 0.166; t(27) = −5.74, p < 10^−4^, corrected) and V1 – rV4 (free view, z = 0.224 ± 0.136; search, z = 0.363 ± 0.137; t(27) = −5.253, p < 0.001, corrected; Fig. 3B). In contrast, decreased correlations were found for V1 – lPIT (free view, z = 0.044 ± 0.122; search, z = −0.117 ± 0.161; t(27) = 4.416, p = 0.003, corrected), lV4 – lPIT (free view, z = 0.082 ± 0.120; search, z = −0.043 ± 0.199; t(27) = 3.505, p = 0.034, corrected), and lV4 – rPIT (free view, z = 0.098 ± 0.120; search, z = −0.040 ± 0.156; t(27) = 4.876, p < 0.001, corrected). Notably, the V1 – lPIT revealed a significantly negative value across subjects (one-sample t-test, p < 0.001) for the search condition, indicating that V1 and lPIT were inversely engaged during searching for a target object (anti-correlation). The other decreased correlations of lV4 – lPIT (p = 0.263) and lV4 – rPIT (p = 0.192) were not significantly different from zero. This connectivity structure suggests a division of the visual ventral stream and a functional boundary was located between PIT and V4, distinguishing the high-order visual area PIT from those of lower and intermediate areas (V1, V4). Further confirming this notion, a liberal p-value of uncorrected 0.05 (free view vs. search) was applied and it added a decreased correlation of rV4 – rPIT (free view, z = 0.038 ± 0.099; search, z = −0.022 ± 0.110; t(27) = 2.329, p = 0.028) and an increased correlation of lV4 – rV4 (free view, z = 0.408 ± 0.148; search, z = 0.470 ± 0.161; t(27) = −2.162, p = 0.040). This pattern of connectivity was also observed when the search condition was compared with the memory condition (Fig. 3C), suggesting that the division was specific to search as compared to the memory and free view conditions. In contrast to the search condition, we observed no anti-correlation of the means across subjects for any ROI pairs in the free view and the memory conditions (free view z > 0.028; memory z > 0.030). Instead, relative to the free view, the memory condition showed an increased correlation of rV4 – rPIT (memory, z = 0.121 ± 0.111; t(27) = −3.436, p = 0.040, corrected). A liberal p-value of uncorrected 0.05 added a decreased correlation of V1 – rV4 (memory, z = 0.161 ± 0.146; t(27) = 2.303, p = 0.029). Therefore, contrary to search, memorizing objects appeared to be related with a strengthened connection of the intermediate area V4 with the high-order visual area PIT, but weakened connection with the lower-order area V1.

As significant activation clusters were also observed beyond the visual areas (see Table 1), we further examined the interactions with these clusters, i.e., the left and right supramarginal gyri (lSMG and rSMG), left and right frontal poles (lFP and rFP), and the right precentral gyrus (rPCG). The lSMG (2067 voxels) covering the left angular and postcentral gyri was defined by the joint effect of the five ROIs’ seed-based maps (V1, lV4, rV4, lPIT, and rPIT), since all these ROIs revealed a significant task-dependent correlation with the lSMG (Table 1). Likewise, the rSMG (384 voxels) was identified by the joint effect of the four ROIs’ seed-based maps (lV4, rV4, lPIT, and rPIT). On the other hand, the lV4 seed-based map identified significant task-dependent correlations with the rPCG (124 voxels), lFP (251 voxels), and rFP (134 voxels). ROI-to-ROI correlations across all 10 ROIs (V1, lV4, rV4, lPIT, rPIT, lSMG, rSMG, lFP, rFP, and rPCG) were separately subjected to a repeated measures ANOVA and revealed 22 significant task-dependent correlations out of 45 possible pairs (Table 2; p < 0.05; Bonferroni corrected for 45 multiple tests), retaining the seven correlations found in Fig. 3. Notably, during the search condition early and intermediate visual areas (V1, V4) were anti-correlated with all the five ROIs beyond the visual ventral stream (lSMG, rSMG, lFP, rFP, and rPCG), further supporting the distinction of V1 and V4 from the higher-order brain areas. In contrast, the memory condition increased the correlation between rV4 and rSMG.

**Table 2:** Task-dependent correlations across ten ROIs.

ROI-to-ROI	Fisher-transformed correlation coefficient (z)		
	Free view	Search	Memory
V1 – lPIT^b,d^	0.044 (0.122)	−0.117 (0.161)*	0.080 (0.134)
V1 – lV4^a,c^	0.299 (0.153)*	0.485 (0.166)*	0.283 (0.200)*
V1 – rV4^a,c^	0.224 (0.136)*	0.363 (0.137)*	0.161 (0.146)*
V1 – lSMG^b,d^	−0.069 (0.149)	−0.247 (0.185)*	−0.051 (0.192)
V1 – rSMG^b,d^	−0.038 (0.108)	−0.116 (0.120)*	−0.000 (0.114)
lPIT – lV4^b,d^	0.082 (0.120)	−0.043 (0.199)	0.066 (0.154)
lPIT – lSMG^a,c^	0.133 (0.144)*	0.293 (0.143)*	0.192 (0.139)*
lPIT – rSMG	0.031 (0.111)	0.125 (0.111)*	0.038 (0.098)
lV4 – rV4^c^	0.408 (0.148)*	0.470 (0.161)*	0.361 (0.121)*
lV4 – rPIT^b,d^	0.098 (0.120)*	−0.040 (0.156)	0.113 (0.128)*
lV4 – lSMG^b,d^	−0.033 (0.136)	−0.251 (0.194)*	−0.013 (0.139)
lV4 – rSMG^b,d^	−0.006 (0.138)	−0.140 (0.132)*	0.029 (0.107)
lV4 – lFP^b,d^	0.034 (0.114)	−0.128 (0.132)*	0.004 (0.089)
lV4 – rFP^b,d^	0.045 (0.093)	−0.069 (0.101)	0.083 (0.114)*
lV4 – rPCG^b^	0.117 (0.124)*	−0.013 (0.148)	0.047 (0.117)
rV4 – rPIT^d,e^	0.038 (0.099)	−0.022 (0.110)	0.121 (0.111)*
rV4 – lSMG^b,d^	−0.030 (0.102)	−0.163 (0.159)*	−0.013 (0.116)
rV4 – rSMG^b,d,e^	−0.047 (0.109)	−0.132 (0.109)*	0.053 (0.112)
rPIT – lSMG^a,c^	0.135 (0.156)*	0.284 (0.155)*	0.158 (0.169)*
rPIT – rSMG^a,c^	0.053 (0.130)	0.177 (0.120)*	0.095 (0.118)*
lSMG – rSMG^a,c^	0.283 (0.173)*	0.394 (0.204)*	0.265 (0.166)*
lSMG – rFP^a,c^	0.122 (0.142)*	0.231 (0.124)*	0.103 (0.143)*

Twenty-two correlations out of 45 possible pairs revealed significant changes across task conditions (p < 0.05/45). Post-hoc tests for each of the 22 ROI-to-ROI correlations were corrected by the same criterion as the strong changes shown in Fig. 3 (p < 0.05/21). The numbers in parentheses are standard deviations of the means. V1 = primary visual cortex; rV4 = right V4; lV4 = left V4; lPIT = left posterior part of the inferior temporal cortex; rPIT = right posterior part of the inferior temporal cortex; lSMG = left supramarginal gyrus; rSMG = right supramarginal gyrus; rPCG = right precentral gyrus; lFP = left frontal pole; rFP = right frontal pole. ^a^significant post-hoc results of search > free view; ^b^search < free view; ^c^search > memory; ^d^search < memory; ^e^memory > free view; *p < 0.001 (two-side one-sample t-test across subjects).

### ROI activity

**Figure 4:**
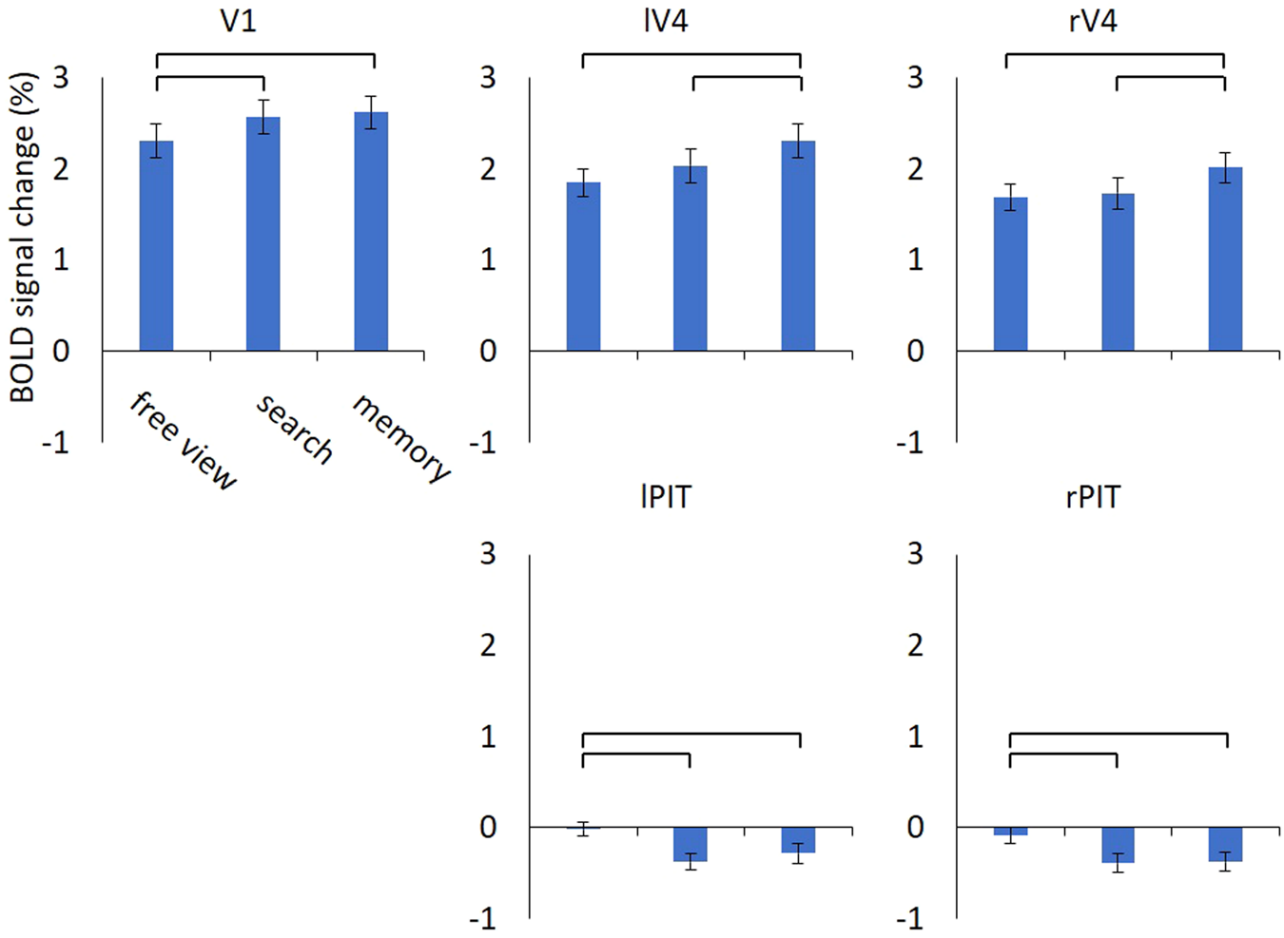
Average signal changes in response to the scene images. The V1, lV4, and rV4 showed task-related activation, while the lPIT and rPIT showed task-related deactivation particularly during the search and memory conditions. Repeated measures ANOVA revealed significant task effect on all regions (all p’s < 0.05, corrected). Error bars are standard error of the mean, whereas horizontal bars indicate significance in the paired t-test between conditions (all p’s < 0.05, corrected). V1 = primary visual cortex; rV4 = right V4; lV4 = left V4; lPIT = left posterior part of the inferior temporal cortex; rPIT = right posterior part of the inferior temporal cortex.

We further assessed the instantaneous BOLD signal changes in response to the scene images, instead of interaction between ROIs. Individual mean BOLD activity in each of the five ROIs was subjected to a repeated measures ANOVA with task condition as a within-subject variable. A significant task effect was found for all ROIs (all p’s < 0.001; Bonferroni corrected for five multiple tests; Fig. 4). Post-hoc analyses revealed that, for V1, the search and memory conditions increased BOLD activity than the free view condition (all p’s < 0.003, Bonferroni corrected for three multiple tests). On the other hand, the memory condition showed the highest activity in rV4 and lV4 than the other two conditions (all p’s < 0.001, corrected), whereas the free view and search conditions did not show a significant difference (all p’s > 0.139, corrected). In contrast, rPIT and lPIT decreased activity during the search and memory conditions as compared to the free view condition (all p’s p < 0.007, corrected), whereas the search and memory conditions were not significantly different (all p’s > 0.784, corrected). All the activity was significantly different from zero (all p’s < 0.020) except for the lPIT and rPIT during the free view condition (all p’s > 0.363).

## Discussion

The seed-based approach allowed us to identify a task-dependent functional network, by explicit comparison of seed-to-voxel correlations during the free view, search, and memory conditions taken on identical visual stimuli. The connectivity across the identified ROIs was organized into correlated and anti-correlated structures according to the context of visual cognition. Searching for a target object distinguished the visual areas V1 and V4 from the high-order visual area PIT, whereas memorizing objects strengthened the connectivity between rV4 with rPIT. Furthermore, task-dependent activation was found in V1 and V4, while PIT showed deactivation during the search and memory conditions.

Anti-correlated BOLD activity has been observed between networks, namely between the regions exhibiting task-dependent activations and deactivations^18,19^. A set of brain regions that are activated during task performance, known as the task-positive network, manifests strong correlations between regions. The correlated brain regions are considered to serve an integrative role in combining neural processes and thus facilitating the execution of a task. In contrast, a set of deactivated brain regions that are anti-correlated with the task-positive network is thought to operate as segregating neural processes and subserving competing or inhibitory representations, known as the task-negative network^20^. The same mechanism seems to hold during the search condition, in which the search condition showed high BOLD activity at V1 and V4 with strong correlations between them. On the contrary, the deactivated region left PIT was anti-correlated with the activated region V1. In this regard, the presence of anti-correlations indicates a dichotomy between low and high-order visual areas, which may have served a competition or inhibition of neural processes. The task-dependent activation suggests that low-order visual area V1 and the intermediate area V4 were strongly involved in searching for a target object, while the high-order area PIT was suppressed.

Our results also showed an increased correlation between regions that exhibited task-dependent activation and deactivation. During the memory condition, the activated region right V4 was strongly correlated with the deactivated region right PIT. It is possible that although the extent of task-dependent activity was different between these two regions, the fluctuations of the BOLD time-series were synchronized. Indeed, this association has been reported in a previous study^21^, suggesting that a brain region that does not exhibit significant activation may still contain a population of neurons that communicate with other regions. Therefore, task-dependent activation at V4 with the presence of deactivation at PIT during the memory condition suggests that, though speculative, memorizing objects might recruit rV4 and communicate with the high-order area rPIT other than the low-order area V1, as we observed a decreased correlation between rV4 and V1.

Importantly, the increased and decreased relationships of the search condition distinguished the V1 and V4 from PIT. Besides, the increased correlation found during the memory condition between the right V4 and PIT highlights the significant role of the division during the present task conditions. Several lines of evidence have shown that as signal moves from V1 to V4 to PIT, the onset latency, receptive field size, and the neural selectivity for complex shape are gradually increasing (for reviews see refs^2,22^). Thus, object representation at high-order areas such as PIT is considerably complex than at low (V1) and intermediate (V4) areas. Given this nature, one reasonable interpretation of the connectivity structure is that during the search condition subjects selectively focused on relatively simple features, whereas during memorizing their focus was directed to features that are more complex. It should be noted that recognition of a specific object might require more than low-level features such as line and orientation^6^, in which most of the objects share these low-level features. Therefore, searching for or memorizing such very simple features is unlikely to be the best strategy for the present task. Instead, our results suggest that the functional properties of V4 such as color, texture, and simple geometric shape^23^ were critical in order to detect the target object, whereas memorizing objects might require much complex object representations.

Since the set-up of the task conditions required subjects to recognize and identify objects on complex scene images, brain responses in the visual ventral stream were expected^2^. However, it does not imply that the brain responses were confined within the ventral visual stream. Indeed, we observed significant task-dependent connectivity with the brain areas covering the SMG, FP, and PCG. Further analyses including all these brain areas highlighted anti-correlations specifically during the search condition, supporting the distinction of V1 and V4 from the higher-order brain areas. This distinction between brain regions may indicate segregated brain networks that are functionally optimized for searching a target object, whereas memorizing unspecified objects might have requested increased integration across brain networks. One critique of the present task paradigm would be that the search condition compared with the free view and memory conditions required a different sequence of presentation, i.e., a target object was initially presented before the scene image in the search condition. This could have required processing resources such as working memory at the initial stage of the search trial, which might have influenced brain responses during the scene image presentation. Future studies with a careful modification of the task paradigm, for instance, comparing simple with complex target object presentations, may prove useful in extending the findings of the present study.

In summary, the present study demonstrated different functional structures of the visual ventral stream. In particular, while the ventral stream was organized into correlated and anti-correlated structures during searching for a target object, memorizing objects manifested a correlated structure. Our results further suggest a putative boundary between V4 and PIT, which partitions the visual hierarchy into two subdivisions that interact competitively or cooperatively depending on task demand. These results highlight the context dependent nature of the visual ventral stream and may provide theoretical and computational pursuits of finding optimal structure in a hierarchical system.

## Supplementary information

supplementary information

## Data Availability

Data are available from the corresponding author, upon reasonable request.
