# Supplementary material for: Task-dependent functional organizations of the visual ventral stream
Source: Sci Rep. 2019 Jun 27;9:9316. doi: 10.1038/s41598-019-45707-w (PMC6597703; doi:10.1038/s41598-019-45707-w)
Supplement: Supplementary file 1 — supplementary information [file 41598_2019_45707_MOESM1_ESM.pdf]

SUPPLEMENTARY INFORMATION

Task-dependent functional organizations of the visual ventral stream

Han-Gue Jo[1,2,*], Junji Ito[3], Barbara Schulte Holthausen[1], Conrad Baumann[4], Sonja Grün[3,5], Ute Habel[1,2], Thilo Kellermann[1,2]

1. Department of Psychiatry, Psychotherapy and Psychosomatics, Medical Faculty, RWTH Aachen University, 52074 Aachen, Germany

2. JARA-Institute Brain Structure Function Relationship (INM-10), Research Center Jülich and RWTH Aachen University, 52074 Aachen, Germany

3. Institute of Neuroscience and Medicine (INM-6) and Institute for Advanced Simulation (IAS-6), Research Center Jülich, 52425 Jülich, Germany

4. Median Klinik Mecklenburg, 19217 Rehna, Germany

5. Theoretical Systems Neurobiology, Faculty I, RWTH Aachen University, 52056 Aachen, Germany

*, Correspondence: hjo@ukaachen.de

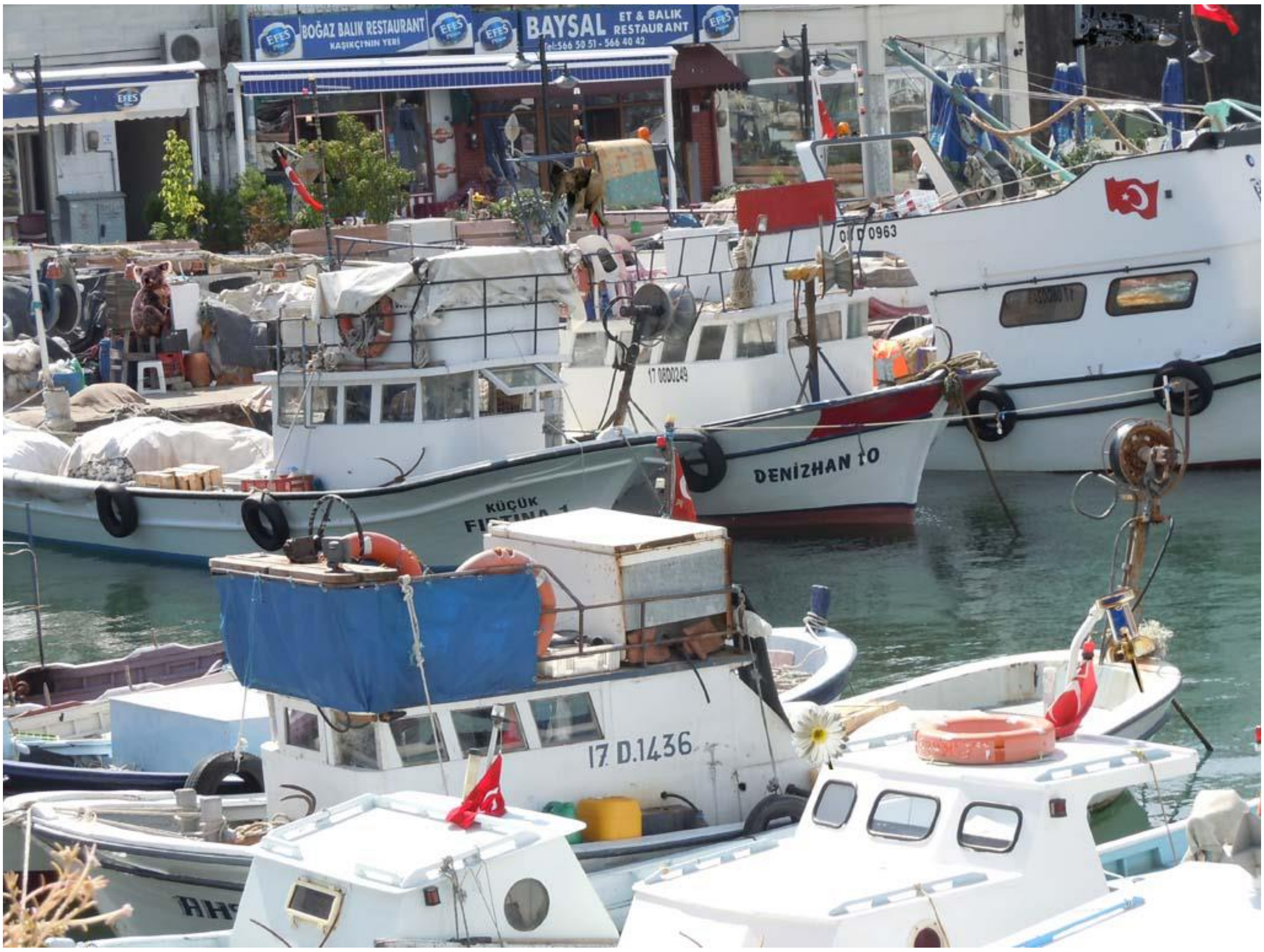

Supplementary figure 1. One stimulus image of a task condition is shown. Koala, train, flower, pin, and elephant are embedded in the scene background image. The background image is a photo taken by one of the authors (MS) of Ito et al. [Sci Rep. 2017 7(1):1082. doi: 10.1038/s41598-017-01076-w], and used with permission. The embedded objects were taken from the Microsoft image gallery.

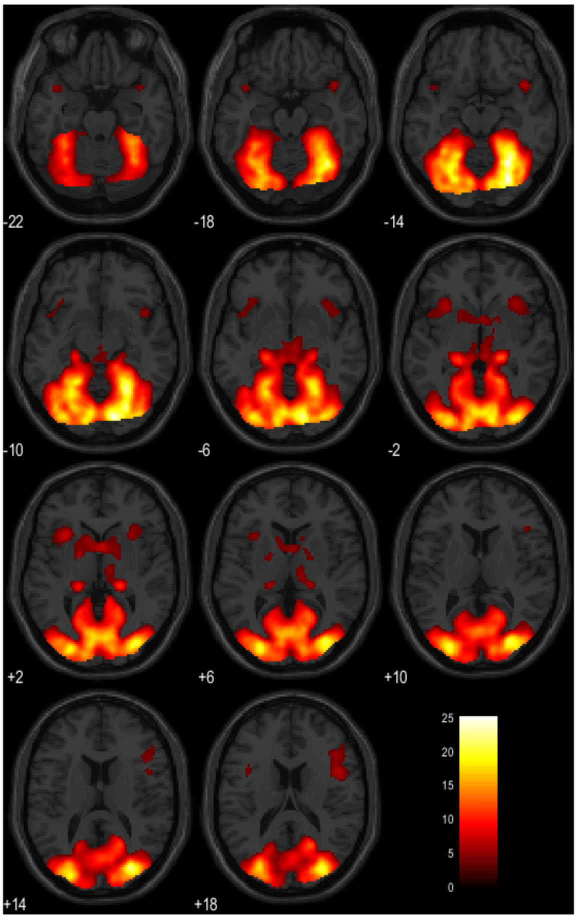

Supplementary figure 2. Group level t-maps from the conjunction of the three task conditions. Significant BOLD signal changes in response to the scene images were thresholded at voxel level uncorrected $p < 0.001$ and cluster-level FEW-corrected $p < 0.05$. The numbers on the bottom indicate z coordinate in MNI space.
